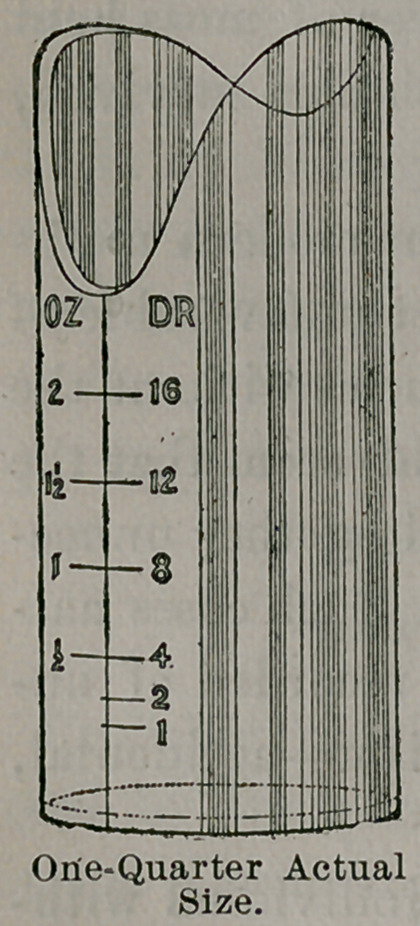# Selections and Abstracts

**Published:** 1901-09

**Authors:** 


### SELECTIONS AND ABSTRACTS.

### Some Surgical Cases.*

By N. SENN, M.D., Ph.D., LL.D.,

Professor of Surgery and Clinical Surgery, Rush Medical College; Attending Surgeon, Presbyterian Hospital; Professor of Surgery, Chicago Policlinic, etc., etc., Chicago.

### CHOLECYSTOTOMY FOR THE REMOVAL OF STONES IN THE GALL-BLADDER.

The first case I wish to exhibit this afternoon is a patient upon whom we operated a few days ago, a diagnosis of gall-stones having been made in the clinic by one of the advisory staff. The clinical history was somewhat obscure, and yet a careful examination of the antecedents of the patient and a thorough physical examination satisfied the consultant as well as myself that it was in all probability a case of gall-stones, of eight years' standing, with repeated attacks. However, only on one occasion, as the history shows, were there indications of jaundice or of obstruction to the outflow of bile. We had reason to assume that we would, in all probability, find in this instance a contracted gall-bladder. We were unable through the intact abdominal wall to palpate the gall-bladder, and it is well known clinically that in cases in which repeated attacks occur, without obstruction to the common or cystic duct, the gall-bladder in consequence of exaggerated physiological function, prolonged for years, becomes contracted or greatly diminished in size. We therefore expected to find a contracted gall-bladder, and a stone in it, or perhaps a number of stones. We performed the operation last Friday. I made the usual incision, parallel with the costal arch directly over the gall-bladder, taking the cartilage of the eighth rib as a central point. On opening the abdominal cavity I found the gall-bladder very much contracted laterally, somewhat elongated, and in palpating it detected a row of gall-stones. The last one of the row, toward the hilus of the liver, was low down. I

*A surgical clinic, held at Rush Medical College.

could move the stones in the gall-bladder very readily backward and forward, but the last stone near the hilus of the liver was immovable. I therefore concluded that possibly this stone occupied the cystic duct. I made taxis, trying to displace the stone from the cystic duct into the gall-bladder, but failed. I then opened the gall-bladder and removed eight gall-stones, all of them faceted, resembling each other very much in size. They were all of unusual size. They were easily removed. I then attempted to dilate the contracted cystic duct. Through the stricture I could detect a stone in the cystic duct by the use of the probe. I finally incised the gall-bladder to introduce my index-finger, and felt the strictured part of the cystic duct, and through the minute opening felt the gall-stone. I attempted to dilate the stricture with forceps, making a number of attempts to do so, but failed to dilate it sufficiently to enable me to deliver the impacted gall-stone. There was only one alternative left, and that was to incise the cystic duct, an operation attended with considerable difficulty owing to the deep location of the site of operation. I made an incision in the cystic duct parallel with its long axis, which was very small, relying on dilatation increasing the size of the opening sufficiently, through which I could extract the stone. The visceral incision was dilated to a sufficient extent to enable me to extract the stone through the opening. I found one of the largest gall-stones in this set impacted in the cystic duct. I then sutured the visceral opening with two rows of sutures, the first row including all the tunics of the duct minus the peritoneal covering, using catgut for the deep sutures. The first row of buried sutures was covered with serous sutures of fine silk, with which the deep row of sutures was safely buried. I sutured the gall-bladder to the parietal peritoneum, inserted a large drain, and closed the external wound up to the point of drainage.

I will pass these stones around for your inspection and examination. They are very interesting specimens, in that they had been in the gall-bladder for seven or eight years. All of them were distinctly faceted, and we have now reason to believe that the clinical symptoms were produced largely, if not entirely, by the gall-stone encysted in the cystic duct, and the remaining gall-stones probably produced no clinical symptoms. The colicky pains were doubtless due almost exclusively to the presence of one of the stones in the cystic duct.

There is one feature to which I desire to refer particularly, after having removed the dressing for the first time, and that is the absence of bile. I informed you at the time of the operation that I found a well defined cicatricial stricture on the gall-bladder side of the encysted stone. I have now reason to believe that there is probably a stricture of considerable extent on the opposite side, which accounts to-day for the absence of bile in the dressings. It is possible that the cystic duct is completely obliterated. If so, at some future time it may become necessary to extirpate the gall-bladder. You will remember that we performed two distinct operations, namely, cholecystotomy for the purpose of removing the stones in the gall-bladder, and a choledochotomy for the removal of the encysted stone in the cystic duct. The patient has had no serious untoward symptoms since the operation, and there is every prospect that she will make a speedy recovery.

### UNILATERAL OOPHORECTOMY AND VENTROFIXATION FOR RETROVERSION OF THE UTERUS.

The next case I show you is one upon whom we performed unilateral oophorectomy and ventrofixation for retroversion of the uterus, but evidences of diffuse pelvic inflammation in the form of extensive adhesions involving both ovaries and the opposite surface of the uterus. I took special pains to fix the uterus in its normal position in such a way that there would be absolutely no possibility of its becoming redisplaced; that is, after the removal of the diseased ovary on the left side I anchored the stump to the abdominal wall and fixed the fundus of the uterus against the abdominal wall above the bladder by two rows of sutures—Czerny sutures and the Kelly suture. The anterior surface of the uterus was sutured to the parietal peritoneum from one cornu of the uterus to the other, while the posterior surface was included by two seizures in one silk suture. At the same time we included the margin of the external incision minus the skin, so that the body of the uterus now rests between two rows of sutures. There will be no possibility of separation of the fundus of the uterus from the location which it now occupies. The wound has healed, as you will notice, by primary intention throughout. We will reseal the wound to-day, reapply a similar dressing, and postpone the removal of the stitches for at least another week.

OSTEOMYLETIS INVOLVING THE METACARPAL BONE OF THE MIDDLE FINGER.

This is the patient upon whom we operated a week ago to-day for a limited central osteomyelitis involving the metacarpal bone of the middle finger. I will remove the dressing to-day for the first time. The disease was characterized by intense pain which was referred to the site of the infection, the central osteomylitis giving rise to considerable swelling outside of the bone; and skiagraph came in this instance to our aid in not only making a pathological but a very accurate anatomical diagnosis. The skiagraph that has been passed around shows near the lower articular end of the metacarpal bone, corresponding to the epiphyseal line, a light spot, which indicates the exact location of the suppurating focus. We suspected in this case a tubercular infection, and we had every reason to believe so from the clinical history of the case. However, the age of the patient, as I informed you at the time, spoke against such a diagnosis, and we found on opening the bone with a small gouge a small cavity in the center of the bone occupied by two or three drops of creamy pus. We made a culture at the time, but I have not yet learned the result. I hope to be able to report it to you at the next clinic. I am satisfied, however, that the culture will show the staphylococcus pyogenes aureus or albus, a microbe which so often gives rise to a limited osteomyelitis. As I predicted at the time, the patient was relieved promptly immediately after the operation, because we succeed in removing the cause of pain—tension. The patient has not suffered since the operation, and I will remove to-day the iodoform gauze drain for the first time. You will note that there is absolutely no indication of suppuration. The wound has healed by primary intention, which is largely due to the thorough mechanical removal of the infected tissue by the use of chisel and sharp spoon, followed by very energetic chemical disinfection of the osteomyelitic cavity. I shall ask the assistant to dress the wound, after the removal of the drain, in the usual manner, and immobilize the hand upon a well fitting anterior splint so as to secure for the hand, and particularly for the finger corresponding with the metacarpal bone, a condition of rest.

HYDROCELE.

This is the case of hydrocele that was operated upon last Thursday by the radical method. I will ask the assistant to remove the dressing to-day for the purpose of inspecting the present condition of the operation wound. You will remember that I performed in this instance a modification of Volkman's operation—that is, I laid open the sac from end to end, which gave me an opportunity to inspect the condition of the tunica vaginalis, and particularly that of the testis. Finding everything satisfactory, the cavity was carefully tamponed with one strip of iodoform gauze, and we took the necessary care to bring the gauze in contact with every vestige of the surface of the tunica vaginalis. That is an important point in the technique of this operation. You will observe to-day that the foreign aseptic substance covers every particle of the tunica vaginalis testis. We are in the habit of removing the packing at the end of five or six days, but a long experience has revealed the fact that unless it is allowed to remain a sufficient length of time occasionally relapse will follow. I shall therefore postpone the removal of the iodoform gauze tampon until the latter part of the week. After having made an incision into the tunica vaginalis, the tunica vaginalis was sutured to the skin with a continuous suture of catgut, and after burying the catgut the approximation sutures were inserted, three or four in number, for the purpose of diminishing the incision to the desired extent—that is, I only left sufficient space through which I could bring out the iodoform gauze tampon. One suture remains in place, which will be tied after the removal of the iodoform gauze packing, constituting as it does, a secondary suture. You will notice that there is very little swelling, no edema, no signs of inflammation. The slight swelling is due entirely to the process of regeneration or tissue proliferation which has been incited by the presence of the foreign aseptic substance in the serous cavity. We will reapply the dressing; dress the scrotum in the elevated position to favor venous return to guard against edema that otherwise might likely occur with the scrotum in the dependent position.

EXTENSIVE VARICOCELE.

I would like to show you this patient a week after operation. It was one of very extensive varicocele. Note the appearance of the scrotum to-day. There is absolutely no edema, no swelling. The transverse wound still remains hermetically sealed with collodion crust, and you will observe particularly the decided shortening of the scrotum on the side operated upon, which is due to the method of suturing the incision transversely instead of vertically. There are no subjective symptoms in this case worth mentioning. The only thing I find objectively at the point of resection is a slight induration marking the exact location of the veins, which were sutured together, so as to restore the continuity of the veins. We shall permit the iodoform collodion crust to remain for three or four days longer, when it, as well as the sutures, will be removed and the line of incision again sealed with collodion.

UNUNITED FRACTURE OF THE CLAVICLE.

This patient was operated on a week ago to-day for ununited fracture of the clavicle. We will remove the dressing at this time for the purpose of giving us an opportunity of inspecting the wound. The patient has had very little pain indeed since the operation. We have removed the fixation dressing, and I will now remove the dressing. I can show you this large wound, which was the seat of decided instrumental manipulation incident to isolation, vivifying, and suturing of the fragments, it having healed throughout beautifully by primary intention. There is no deformity at the site of operation, which is very satisfactory evidence that the sutures so far have done their duty, that the fragments still remain in accurate apposition, and that in the usual course of time we may look confidently for a restoration of the continuity of the clavicle by the formation of bony callus. If this does not take place I shall be disappointed, and in that event we shall attribute the second non-union entirely to the absence of the intrinsic capacity of the fractured bone to produce callus. A week has elapsed since the operation; the wound has healed, hence I shall ask the assistant to remove all the deep sutures, permitting the horsehair stitches to remain in place, and again immobilize the arm and forearm in the usual position—that is, by treating the cavicle in the same manner mechanically as we would a case of recent fracture.

TWO CASES OF FRACTURE OF THE OLECRANON PROCESS.

I want to show you a case of fracture of the olecranon process. The patient was admitted to the hospital some two or three weeks after the accident occurred. The other case is a recent one, the injury having been sustained by the patient last evening. We found on admission of the patient to the hospital in the first case a well marked complication incident to the injury. I exhibited this patient to you on a previous occasion, and spoke of the existence of an extensive swelling of the elbow-joint, most notable at a point above the olecranon process and immediately over the posterior aspect of the lower portion of the arm. The swelling could be directly traced into the joint, as was evident by the apparent widening of the joint incident to extensive intra-articular effusion. I have been able to ascertain through the swollen overlying soft parts distinct evidences of the existence of a fracture of the olecranon process. The injury was followed by considerable intra-articular extravasation. We shall find on opening the joint a considerable amount of extravasated blood. About a week ago I punctured the joint with a small trocar, but failed to evacuate any of its contents, owing undoubtedly to the existence of coagulated blood in the joint. We injected into the joint a three-per-cent. solution of carbolic acid. This intra-articular injection has had a decided beneficial effect. I find the swelling to-day reduced materially in size. I have been hesitating in reference to the time I shall select for the purpose of opening the joint with a view to removing the products of extravasion. But as long as the intra-articular antiseptic injection has had the effect of stimulating the process of absorption, we shall postpone arthrotomy until we become satisfied that the process of absorption has become arrested any time in the future, when we shall not hesitate to interfere promptly. The fracture in this instance is through the olecranon process at a point near its attachment to the shaft of the ulna. I can feel a distinct ridge with a corresponding depression showing slight lateral displacement. However, I am unable to move the olecranon process to any considerable extent, which is perhaps proof that the periosteum has only in part been torn, the olecranon not having lost its entire attacmnent to the shaft of the ulna. We shall immobilize the arm in the proper position, which is that of slight flexion, and I shall instruct the assistant to apply a fifty-per-cent solution of alcohol, which will contribute somewhat toward stimulating the process of absorption. We shall soon resort to systematic massage in this case, another expedient that has a very decided effect in promoting the process of absorption.

The next case is a recent one, the fracture of the olecranon process being caused by a fall from a freight car. The exact manner in which the patient fell is somewhat uncertain, but undoubtedly it was either the result of hyperflexion of the forearm or hyperextension, or the fracture occurred in consequence of the application of direct violence. The patient is able to move the arm to a certain extent, but flexion and extension are greatly diminished. I rely in this instance, in ascertaining the existence of fracture, largely upon the point of tenderness as well as on the preternatural mobility of the olecranon. When I pass my finger along the ulnar ridge I come to a point where tenderness is exquisite. I find now in examining the surface the direct cause of the fracture. There is a hard, well marked abrasion showing where the fracturing force was applied. So in this instance we have positive manifestations of the *modus operandi* of the injury. The fracture occurred in consequence of the direct application of force immediately over its seat, where we find the well marked abrasion I have referred to. I now take hold of the olecranon process, and on attempting to move it laterally I produce pain. I find now that I can move the olecranon process to a considerable extent in a lateral direction, and in so doing I feel very distinctly a crepitus. I have not made extensive manipulations because these would be unnecessary. Having found the line of tenderness corresponding to the fracture, with slight lateral deformity, with lateral movement over the olecranon process, we have positive indications of the existence and exact location of the fracture.

A fracture of the olecranon process must be treated with care, because it means a fracture which involves and extends into the elbow-joint. The only displacement we will find complicating a case where the fracture is complete, with corresponding laceration of the periosteal envelope, is a longitudinal displacement—a diastasis. To overcome this diastasis it becomes necessary to dress the arm in nearly an extended position. I shall direct the assistant to immobilize the arm exactly in such a position, with very slight flexion. This is done by using a straight splint with angular cushion corresponding to the bend of the elbow. The splint is to be held in place by strips of adhesive plaster, one below the thumb, another one above the wrist-joint, and a third one at the base of the arm. Then with a strip of adhesive plaster the upper border of the olecranon process is grasped in the manner I show you; the strip of adhesive plaster is drawn downward and fastened over the splint. We shall not apply this dressing in the clinic for want of time, but I hope to be able to show it to you at the next clinic. The idea is to immobilize the elbow-joint in proper position, the forearm at slight flexion, and to overcome the diastasis directly by making use of adhesive plaster, grasping the olecranon process, and drawing it down in the direction of the shaft of the ulna.

### FRACTURE OF THE SPINE.

I showed you recently a case of fracture of the spine, and now the recovery of the patient is almost complete. I will show you to-day a second case in which there are not only distinct evidences of the existence of a fracture, but its exact anatomical location. The injury was sustained four months ago by hyperflexion of the spine, as is always the case. It is easy to detect the exact location of the fracture at a point corresponding to the tenth or eleventh dorsal vertebra, with marked kyphosis. We have resorted to the following treatment in this as well as in similar cases, when there is no paralysis of the lower extremities: The patient has been placed upon a Rauchfuss splint in the dorsal recumbent position, and this splint is applied both for the purpose of effecting extension of the spine, as well as to secure immobility at the seat of fracture. The fracture has now united firmly. We can at present rely safely upon a fixation dressing, permitting the patient to leave his bed and to walk about in a plaster-of-paris splint, which will be applied in the course of a day or two. I can recommend this splint in the strongest terms in the treatment of fractures of the spine in which there is no paralysis below the seat of fracture, no crushing or compression of the cord causing paralysis of the lower extremities. In case a fracture of the spine is complicated by paralysis, either the result of laceration of the cord or compression from various causes, we make use of the water-bed for the purpose of preventing decubitus. In all other cases the Rauchfuss splint surpasses all other mechanical means of immobilizing the spinal column at the seat of injury.

### FRACTURE OF THE RIBS.

Here is a case of fracture of the eighth and ninth ribs at a point where we confidently can look for a fracture after the direct application of force to the ribs, namely, at the junction of the ribs with the cartilage. There is a separation of the eighth and ninth ribs from the cartilages. I have simply had this patient brought into the clinical amphitheater to show you the method of fixation employed, which consists of circular strips of adhesive plaster for the purpose of immobilizing the injured side of the chest.

### SYNOVIAL TUBERCULOSIS.

You will doubtless remember that at the last clinic we punctured the knee-joint in this case for synovial tuberculosis, and injected iodoform-glycerin emulsion. Unfortunately, the patient was unable to enter the hospital for financial reasons, and has been treated as an outdoor case. I always hesitate to puncture the knee-joints of these patients and make use of such a potent agent as iodoform-glycerin emulsion, for the reason we should have them under direct supervision. The patient expressed his inability financially to take my advice to enter the hospital. I would like to have you notice the size of the knee-joint. We emptied it completely of a copious effusion. After tapping and evacuating the effusion, the knee-joint appeared almost normal, but to-day again it is enormously swollen. I am glad that this is the case, because in all instances when the idoform-glycerin emulsion exerts its therapeutic effect satisfactorily a violent local response takes place. The joint becomes more swollen, as a rule, than it was before the tapping was done. This is the reaction of the tubercular synovial membrane to the agent injected into the joint. There is present now decided effusion. I believe if temperature observations have been made, it will be found that this patient's temperature must have risen at least one to two degrees within twenty-four hours after the injection was made. That is the rule, if the iodoform-glycerin emulsion exerts its curative effect, and the local as well as the constitutional response is always manifested after such operative intervention. We will envelop the knee-joint with a thick cushion of cotton and apply over it a gauze bandage for the purpose of immobilizing the limb, and instruct the patient to report again a week later.

### COLLES'S FRACTURE.

I have shown you many cases of Colles's fracture in the aged during the present winter, but it is not often that I can exhibit to you a case of a similar accident in a child. This boy fell a week ago directly upon the palm of the hand. Note the appearance of the wrist-joint to-day. We have a typical silver-forked deformity in this case, a subluxation of the lower end of the ulna, the seat of the fracture being about three-quarters of an inch above the wrist-joint; deviation of the lower fragment backward toward the dorsal side of the arm; lateral and posterior displacement of the lower fragment—a typical Colles's fracture. I shall recommend that this boy be brought into the hospital. I am informed that only two weeks has elapsed since the accident. If we administer a general anesthetic I am satisfied that we shall be able to break up, by mild manual force, the existing adhesions between the two fragments, effect reduction, and consequently secure a good result. The ulna is not fractured. In the majority of cases in children we find both the radius and ulna are fractured. Here the ulna is intact, but subluxated.

### LARGE INGUINAL HERNIA.

When this patient was lying on the table the hernia could not be very well seen, but the moment he assumes the erect position you will notice the hernia, which is of large size, and descending gradually into the scrotum. I will now ask the patient to cough, and you will notice that the impulse on coughing is very plainly seen. The hernia becomes tense and very much enlarged during the act of coughing. I can easily reduce this hernia with the patient in a standing position. The patient will be advised to enter the hospital for the purpose of having a radical operation performed. The opening is so large and the obliquity of the inguinal canal so much diminished, that in all probability we shall have to make a Bassini operation.

### METATARSALGIA.

This is an interesting case indeed. The patient gives a history of gonorrheal infection, and he now has a symmetrical affection of the metatarsophalangeal joint of the second toe on both feet. In pressing on the dorsal side of the joint I am unable to produce any pain. There is likewise no pain when I press on the lateral side, but there is exquisite pain when I press on the plantar side. Please remember that this is one of those cases which you are apt to overlook. It is not a joint affection, although the patient has had specific urethritis, but it is one of metatarsalgia, an affection first described by Morton, of Philadelphia. He is similarly affected on the opposite foot. That is an important point. This affection is undoubtedly the result of harmful pressure, illy constructed shoes, etc. If this patient will give the feet rest undoubtedly spontaneous recovery will occur. If not, resection of the affected nerve, or even resection of the joint, as has been advocated by Morton, may become necessary to bring about permanent relief; but by physiological rest of the foot in an elevated position I look for a spontaneous recovery in this case.

### GLANDULAR TUBERCULOSIS.

This woman is twenty-three years of age, and states that the glands of her neck began to enlarge about two months ago. When I examine the lower margin of the glandular swelling I am able to detect an additional gland, which aids very much in making an absolute diagnosis. We have here an affection involving at least two glands in close proximity, the smaller one below the larger one. There is not very much pain, and very little tenderness. No fluctuation. It is a plain case of glandular tuberculosis, for which excision will be urgently recommended.

### TYPICAL BLACK EYE.

I want to show you a typical black eye in its early stages, such as results from a fall or a blow. To-morrow morning this woman's left eye will be nearly closed, and the characteristic black discoloration of the skin will take place. She fell directly upon the superciliary arch. I wish to call your attention to the subcutaneous extravasation here. There are doubtless two teaspoonfuls of blood between the bone and the pericranium. I can palpate through the orbital arch very distinctly; consequently I believe that this extravasation is between the bone and the pericranium. We shall recommend compression and the application of some resorbent stimulating lotion, such as a 25 per cent. solution of alcohol, or a solution of muriate of ammonia combined with well applied, well fitting compression—agencies resorted to for the purpose if expediting the removal of the extravasation by absorption. I could, of course, insert a small trocar and remove the extravasated blood, but the same thing can be accomplised in two or three weeks by well applied compression and the use of local remedies calculated to promote the process of absorption.

### MASTOIDITIS

This patient I have exhibited to you before. It was originally a case of otitis media with mastoiditis, for which an operation was performed outside of the hospital. Permanent facial paralysis followed the operation, and the patient has only received partial relief from the intense, agonizing pain, which he refers to the temporal region largely, as well as to the region immediately above the external ear. I want you to be as quiet as possible, so that I can demonstrate a very important physical sign. By tapping over the temporal region I am able to elicit marked tympanitic resonance. It sounds like tapping on a drum. By tapping over the temporal region of the opposite side we get the very opposite of tympanitic resonance; it is solid. This tympanitic resonance, as I informed you before, is due to extension of the infection from the middle ear to the meninges of the brain, of which we now have positive evidence. I have no doubt that in operating on this patient we shall find putrefactive bacilli and extremely fetid pus.—*Medical Age.*

---

### BURDETTE'S ORATION AT THE CALIFORNIA DENTAL CONVENTION.

At the closing session, July 11th, of the Dental Convention, Rev. Robert J. Burdette gave the following amusing oration as quoted from the Los Angeles *Times.*

"Man that is born of a woman is of little hair and no teeth when he is born, and sometimes it would be money in his pocket if he had less of either," said Rev. Mr. Burdette.

"As for his teeth," he continued, "he hath recurring convulsions when he cuts them, successive toothaches so long as he hath them, and as the last one is coming through the first one is falling out; and he entereth the afternoon land of his days, a human machine, having a mouth full of porcelain teeth built upon a plate that is constructed to hold raspberry seeds, so that the last state of that man is worse than the first. [Laughter and applause.]

"Even so if he shall stand up in the glory of old age and say, 'I am a true man,' he is condemned out of his own mouth, for molar crieth unto incisor, 'Thou liest in thy teeth.' Happy is he if he possess the teeth that have the cheek—though not the nerve—thus to reproach him.

"Much honored is any man to stand before this assembly; the representatives of a profession, whose work, since the conventionalities of civilization have abolished the custom of scalping, stands at the head of all surgery. Like the sun-dial, your work marks only the smiling hours. The rest of us conceal our shortcomings; we hide our mistakes; we deny our infirmities; while you, oh, fearless, honest men, you glory in the display of your 'false.' This is, indeed, tooth in. [Laughter and groans.] I do not wonder that you groaned. The rest of us groan when its tooth out.

"Shakespeare, whose genius transcends mere human culture, could exalt the toothache and never lose a note of grandeur. In his words I glorify your profession, for

> " 'Your desert speaks loud, and I should wrong it,
> To lock it in the wards of covert bosom,
> When it deserves, with characters of brass,
> A forted residence against the tooth of time.'

"You see, Shakespeare evidently knew nothing about gold filling. But he knew what toothache was. We have internal evidence for that. He recognized its grandeur of anguish, its titanic potentiality of pain. He used it as a simile for the deepest and most distracting throes of human agony and rage of grief.

"Shakespeare never treats the toothache lightly nor irreverently, after the shallow fashion of the every-day humorist. In 'Much Ado About Nothing,' when poor old Leonato is heart torn in an agony of grief and shame, bitterer than death, in the wildness of his rage and suffering, spurning the sympathy of his friends, he cries:

> " 'I will be flesh and blood,
> For there was never yet philosopher
> That could endure the toothache patiently,
> However they writ the style of gods,
> And made a push at chance and sufferance.'

"Shakespeare never repeats, therefore, when thrice he uses the toothache as a figure of profoundest suffering that can rack mind and body, we know with what reverence and gratitude this immortal man would have dedicated the work of his pen to the California Dental Association. [Applause.]

"Under the old Hebrew law, 'If a man smite out his servant's tooth, he shall let him go free for the tooth's sake;' that was the value of a single tooth—the whole man. [Laughter.]

"Well may the dentist wear his crown of gold upon his patient's teeth; for right royal is he in pedigree and fame. Whatever he does he does sublimely. When he harpoons a hysterical nerve to see if it be alive, he leaves no doubt in the mind of the patient, the ears of the neighborhood, or the duty of the Recording Angel, that the nerve and the patient's organs of phonation are as much alive as they are sadly out of tune.

"We admire him as a calm and progressive corrector of human evils as we view him putting a gold filling in Mr. Bryan's wisdom tooth, or filling the mouth of a preacher with a rubber dam—the only kind that preachers and dentists—I speak under correction of the dentist—are permitted to use. And that, too, is in keeping with the time, for theology of to-day is nothing if it be not elastic. [Laughter and applause.]

"If you bring a brand new piece of humanity to the dentist, a dimpled baby, with the gummy, toothless grin of infantile happiness wrinkling its downy visage, it wakens no professional interest in him. It is too new. By and by, when there are repairs to be made, the mother brings the little one to the high chair behind the screen. Nature, and the physician, and the nurse, and the minister who christened the child, have all done their best. The little human machine has been fairly started on its seventy year run, and it hasn't run ten miles before it must go to the repair shop. The higher the civilization the greater the strain upon the machine. Something to be braced; something gone awry that must be straightened; something gone so loose that it must be removed; civilization to be rebuked and nature to be corrected; and the dentist repairs and corrects the mistakes of nature and civilization, peaceably as he can, forceply if he must. The more delicate the machine the more need of continuous repair. We must in all honesty and the highest appreciation, exalt the repair shop.

"And of all dental repairers on earth, the American stands not only at the head of the highest class, but he stands in a class alone—without competition outside of his own country. [Applause.] The highest praise ever accorded to the American dentist we heard in the cities of Europe last year. Some repairs were necessary in the mills which for many months had been grinding the vulcanite steaks of Italy, and fracturing the flinty relics of the stone age which the French people are taught to call bread. [Laughter.] We found in a city of Switzerland a promising sign in blue and gold—'American Dentist.' That was what we wanted. We climbed the stairs hand in hand—nobody ever goes alone to a dentist's—whispering words of encouragement and cheer to each other. We entered the sanctuary. A man bearded to the eyes, saluted us in German. 'Do you speak English?' I asked. 'Nein,' he replied. 'Sprachen sie Deutsche?' 'Nixie weeden,' I said, and we left 'the American dentist' waiting for customers who could speak German. [Laughter.]

"Much do I wonder that I, who should have been the star subject at the clinics, should appear before this learned body of professional men as an orator. For I am not a man in whom the dentists take delight, however much they may regard me as a curious and interesting study. I have no doubt that the thought that flashed into the mind as this speaker confronted you, whistling his words with painful effort through the waste spaces where the teeth used to be, many years ago—a mocking reproach to dental cunning and learning—was 'an enemy hath done this thing,' Yet I am not here to mock you. [Laughter.]

"I was about to say that it was not my fault that I do not smile down upon you in the glittering grace of hard finish porcelain. Like the woman in the Scripture 'who had suffered many things of many physicians, and had spent all that she had, and was nothing bettered, but rather grew worse,' so I have writ my experience with the dentist. Each successive man to whom I went, praying for more teeth, not only refused to give me that for which I asked, but took away at least one of the teeth I had. Oh, some of the more hopeful ones tried. [Laughter.]

"I have carried misfit plates in my pocket, where, as I moved about, I could hear them snarling and biting each other in professional jealousy. [Laughter.] But gradually the verdict of united dentistry became unanimous. They said my mouth was not made right. It would not fit any plate that human skill and dental science could shape. I said that my mouth was made first, and the plate should fit my mouth. They insisted with many long and impressive words, that my mouth did not fit. [Laughter.]

"One or two cheerful practitioners did offer to remove every tooth left, saying they could do something for me if they started in with an entire outfit. But this was so much like building a new barrel around an old bunghole, that I hesitated. [Laughter.]

"And for years I have gone up and down the land eating my bread in the sweat of my mouth, making my living with my degenerate jaws, counting my few remaining teeth every morning to see that none had been captured or added to the death roll, for I knew that as the ranks of the Old Guard became thinned, there could be no recruit to take the place of the captured veteran. [Laughter and applause.]

"And when in far-away Syria, there came the request that I should deliver an address before the California State Dental Association, my entire household woke the echoes of the Jordan Valley with inextinguishable laughter. The very Arabs of the camp enjoyed the humor of it with unbroken sets of the pearliest teeth that ever lighted up a laugh. A little flame of wrath smoultered and flared, red and fitful under my mirth. I said:

"'These dental Philistines—accent heavy on the Phil—they have shorn Samson of his locks; they have put out his eye-teeth; and, now, when their hearts are merry in the house of Dagon, they say, "Call for Samson that he may make us sport."' And you remember how excruciatingly funny Samson was. He brought down the house. [Laughter.]

"Ah, well; the dental excuse—good excuse—for my impossible mouth is a good one, whether it be valid or no. I, too, make frequent use of it. Whenever, after trying my best through a long evening of mirth to bring a smile to some hardened countenance in the audience, grim, stolid, inflexible, hopelessly stupid, incurably asinine, the fixed degeneration of clammy imbecility, I say: 'The fault is not with me, nor with my oft-repeated jokes; it is the man; he is devoid of the sense of humor.' And he grieves me no more.

"This fun of ours is all in the family, oh, brother dentists, for I am one with you and one of you. I, too, love a genial display of teeth, hand-made or natural; perfect in surface or showing the radiant gleams of costly filling. I, too, am a jawsmith. [Laughter.]

"'But,' says the critical and professional listener, 'this speech of yours does not fit the subject.' Oh, my brother, remember what you said about my mouth—your subject does not fit my speech." [Laughter and long-continued applause.]

---

### THE INCREASING STERILITY OF AMERICAN WOMEN.*

This investigation is based upon numbers which may seem small to admit of deductions as to conditions existing throughout a great country, but I feel justified in doing so as the data are exact and cases are carefully sifted, in addition, many details are corroborated to a decimal by independent observers in far-distant points, first and foremost by the census records of two great States—by Dr. Wilbur, in the census of Michigan, and by Drs. Abbott and Kuszynski, in that of Massachusetts; by the careful observations of Dr. Chadwick, in Boston, and for the eighteenth century by town records from Massachusetts communities. Certain data are taken from each, as no one investigation covers all the points I have developed, and some have never before been presented, so that no record for comparison exists; all are indirectly corroborated by correlated facts. Whatever may be thought of the results obtained, the data presented certainly suffice to indicate the imperative need for further and more extended investigation in this direction.

The sterility of woman has increased, hand in hand with the much-discussed decrease of fecundity, everywhere to some extent but in the United States to an excessive degree, as fecundity has diminished more rapidly than in other countries—from a sterility of 2 per cent. in the eighteenth century and a fecundity of five children to the marriage, conditions better than in any other country and such as led to the Malthusian theory of super-fecundity, to the fear of over-population of the earth's surface, after a lapse of one century *from first* we have passed *to last* and the other extreme is now presented—*sterility greater* and *fecundity less* than that of the women of any other nation, unless it be of France, who for this reason must yield her proud position of one-time supremacy and retrograde to the rank of a second-class power.

Among the laboring class in St. Louis, 21 per cent. of all mar-

*Read by Geo. J. Engelmann, M.D , Boston, Mass., before the Gynecological Section of the American Medical Association, St. Paul Meeting, Jnne 4-7, 1901. Author's Abstract.

riages are sterile; 24 per cent. among the higher classes; of foreigners, only 17 per cent.; throughout the State of Massachusetts, Americans 20.2 per cent., foreigners 13.3, and in the city of Boston, the laboring class, American born, 23.1 per cent.

Among the laboring class, American born, the fecundity in the eighteenth century was five children to all marriages; at the beginning of the nineteenth century, 4.5; it was at the end of that century 1.8 to 2. Two in Missouri, 1.8 in Michigan, 1.8 in Boston; somewhat more among American born of foreign parentage, much more among foreigners; among the Irish 4.2 in St. Louis, 3 in Boston, 5 in Michigan; among the Germans 3.4 in St. Louis, 6 in Michigan, and in Massachusetts for all foreigners 4.9 children to the marriage. Fecundity somewhat less among the native American, also among the higher classes, least of all among college graduates, 1.6 children to the married couple; in England 1.5, whilst for the population at large it is 4.2.

I have called attention to the frequency of miscarriage and divorce as concomitants and causes of sterility, mainly to emphasize that barrenness is not due to physical causes, to pelvic disease amenable to local treatment, and that sterility is but too often the sequence to intentional miscarriage and the methods which precede it, the prevention of conception, both of which competent investigators have shown to be far too frequent.

Divorce in Canada is 1 to 63,000 marriages, in England 1 to 11,600, in Germany 1 to 13,000, in France 1 to 12,500, in all the United States 1 to 185; in Massachusetts 1 to 18.8, Rhode Island 1 to 8.2.

Miscarriages are found in the proportion of 1 to 2.8 labors at term among Americans; 1 to 5.5 is the usually accepted standard. Among Americans of American parentage the frequency is somewhat greater, 1 to 2.7; among American-born of foreign parentage somewhat less, both in St. Louis and Boston, 1 to 3; among negroes worse.

There is an absolute and primary barrenness due to utera-ovarian disease, mainly to atresia, gonorrhea and to endometritis, with acrid discharge, destructive to the spermatozoa; this is here for the first time clearly distinguished from relative or secondary sterility, *i. e.*, conception and miscarriage; this primary sterility is much less frequent, 12 per cent. among Americans, 10 to 11 per cent. among foreigners, which of course means relative sterility for Americans 9 to 12 per cent., for foreigners 3 to 6 per cent., showing that among American-born there is a much greater proportion of sterility, of childlessness, due to abortion; this may be due to disease or accidental traumatism, more often authorities say not. Much of the barrenness of women is intentional. All sterility in the American colonies was 2 per cent., in parts of Russia to-day 2.8 per cent., in Norway 2.5; hence primary sterility can certainly, in this country, not be over 8 per cent.; 8 per cent. of 20 to 23 of the childless, and even absolute, primary (by barren marriages) sterility is, once in four or five cases, due to the male, showing that absolute sterility in woman is not common and that sterility is not mainly due to utero-ovarian disease; this, moreover, is evident from its rapid increase, hand in hand with the astounding progress of gynecological science, which we have every reason to believe would reduce the number of childless women to a minimum were sterility referable to tangible physical causes.

Sterility is a sad affliction for the innocent sufferer, and for her our best efforts must be exerted; but if so rarely due to pelvic malformation and disease why do I present these thoughts to the Gynecological Section of a medical society? It is because the subject is a pertinent one to us as men, as physicians, if not as gynecologists; it is because we must seek to stay the progresss of this abnormal state—because men and women are in ignorance of the suffering prone to follow willful and self-inflicted sterility; and it is this subject which claims a prominent chapter in the gynecology of the future, in preventive gynecology.

The death-rate of nations has steadily decreased in the last decade by the development of preventive medicine, and so may sterility decrease and birth-rate increase with the progress of preventive gynecology.

### PUERPERAL SEPSIS.*

In the essay of Oliver Wendell Holmes on the "Contagiousness of Puerperal Fever," reference is made to the fact that in 1795 Doctor Gordon, of Aberdeen, published a treatise on the same subject, submitting proof of its infectious nature, and saying, "I arrived at that certainty in the matter that I could venture to foretell what women would be affected with the disease upon hearing by what midwife they were to be delivered, or by what nurse they were to be attended during their lying-in; and in almost every instance my prediction was verified." At this time the profession was almost a unit in ascribing the disease to visitations of providence; and to Dr. Gordon principally is due the credit of having launched that great truth, which, largely through the efforts of Holmes and Semmelweis, has become universally accepted, and which ascribes the disease, not to the "visitations of providence," but to the visitations of man.

Knowledge of its source led to the knowledge of its prevention. Practitioners no longer carried around, in their coat-pockets, infected uteri, obtained at autopsies, and they became more and more generous in the use of soap and water, until, at length, a great advance was made by Semmelweis in his advocacy of solution of chlorid of lime for cleansing the hands. Finally, present methods, initiated by Lister, leave, it should seem, no excuse for series of cases in the practice of any practitioner. Yet such occur. Within a year I have been informed by a reputable practitioner that one of his colleagues had ten cases in as many successive lying-in patients. Knowledge of this fact alone induced me to accept appointment on this committee; and of itself it is a sufficient reason for appearing before you with a subject to which I cannot hope to add to the sum of our knowledge a single item.

Isolated cases appear in the practice of the most conscientious and painstaking; but my own observation, coupled with the reports which appear so frequently in the journals, has convinced me that puerperal sepsis occurs with unjustifiable frequency, and the responsibility must rest mainly with the medical attendant,

*Read by John Fife, M.D., Red Bluff, before the Medical Society of the State of California, April 16-18, 1901.

for his duty is not limited to efforts to render himself incapable of conveying infection ; he must assure himself, with reasonable certainty, that the nurse adopts proper precautions, and that the surroundings are as clean as it is possible to make them. The weight of responsibility in these cases with the general practitioner in the country is great. His field frequently covers a large territory, and he is generally compelled, in the outlying districts, to intrust the entire after treatment to the local "midwife," who is usually armed with a syringe, which she views as a panacea for the ills of parturient women, and who knows at once too much and too little. Even at home he can rarely command the services of a trained nurse, and he is perforce compelled to exercise personal supervision of the after care of the patient.

Preparation of the patient has occupied much attention, and varies from the simple bath to the elaborate measures of the Dresden school. The observations of Williams and Kronig have shown that pathogenic germs do not exist in the vagina, whose secretions, it is believed, possess germicidal power, except as to the gonococcus. Not so the vulva and the vulval canal. Here, according to Edgar, in a proportion of cases pyogenic bacteria are found. It should seem, therefore, that preparatory scrubbing and douching of the vagina with antiseptic fluids is unnecessary, and consequently unwise, and that the bath, followed by scrubbing of the external parts, and sponging off the vulva and vestibule with an antiseptic solution, will fulfill all of the indications. Edgar insists in addition that, if possible, in introducing the sterile fingers, these parts be not touched by them. But the most important measure by far consists in the practitioner's ridding himself of infectious agents. It may be affirmed with confidence that at the present time, as in the past, the vast majority of puerperal fever cases are traceably to neglect of this important measure. The medical world is agreed that, in these cases, as a rule, infection comes from without; yet practitioners who resort to the most elaborate preparation for an important surgical operation are content, in obstetrical work, to give their hands a "lick and a promise" with soap and water, swash them with bichlorid solution, dip them in doubtful olive oil, and proceed to the examination. Surely exercise of the plainest common sense should point out to them their inconsistency. If records of the lying-in cases of these gentlemen were obtainable, they would undoubtenly show a high rate of morbidity, as well as of mortality, though the excess perhaps might not be ascribed to sepsis. But there is more in a name than Shakespeare would have us believe.

With the people "child-bed fever" and carelessness are often viewed as effect and cause, sometimes unjustly; and hence, it may be, malaria and typhoid, already heavy laden, have additional burdens thrust upon them; for, under these names, responsibility for sepsis may still be shifted to providence. Except in the presence of great emergencies, as, for example, severe post-partum hemorrhage, where there can be no question as to the choice of evils, the practitioner should prepare his hands with exactly the same care in obstetrical as in surgical work, and he should avoid soiling them with infectious material in his daily rounds. He who has had occasion to remove a decayed placenta without gloves has been taught by experience how difficult it is to free his hands from the odor. The vigorous use of soap, brush, and hot water for thirty minutes, followed by another brush, and bichlorid, carbolic, or chlorid of lime, for an additional thirty minutes, will not do it. But, after prolonged effort, his olfactories may convince him that he has succeeded. Let him place his hands in front of the nose of his dog, who is peculiarly attracted by vile odors, and the interest he (the dog) displays will at once undeceive him. And that it is probably not less difficult to remove cause than effect, I may cite the well-known fact that the microscope and the culture tube have shown that the most elaborate methods of hand disinfection frequently fail of their object. The importance of avoiding soiling the hands with infectious material, at all times, is therefore apparent. The clothing should not be overlooked. Notwithstanding the recent assertion of a New York sanitarian, that infection is rarely, if ever, conveyed by this means, the wise man will continue to change his clothing after visiting patients with smallpox, diphtheria, and other infectious diseases; and he will be no less careful in his attendance upon lying-in women not to wear clothing that may possibly have been soiled while attending autopsies, incising phlegmons, erysipelatous abscesses, etc.

In estimating the value of any special plan of treatment of this disease, three points should be maturely considered, namely, the degree of individual susceptibility, the variety, and the degree of virulence of the causative germs. As is well known, different individuals differ in their powers of resistance to disease; saprophytic is much less formidable than streptococcal infection, and the type of the disease differs at different times. Hence has arisen diversity of opinion regarding special methods of treatment. The same method may alternately be lauded by the many and condemned by the few, and condemned by the many and lauded by the few. All this will change only when we have reasonably exact knowledge of first causes.

Curettage, once highly esteemed, and still in vogue with many, is gradually being discarded as inefficient and mischievous—inefficient in that it fails to move the cause, and mischievous in that it opens up new avenues for infection and breaks down the barriers nature has erected against her invaders. Intermittent flushing of the cavity of the womb, as Pryor states, cannot reach the cocci in the deeper structures; but I believe he underestimates its value and exaggerates its dangers.

Antistreptococcus serum has been a disappointment, possibly because there are streptococci and streptococci, which, in the present state of our knowledge, are indistinguishable, but which differ in the character of the toxins they excrete. I shall pass over inunction of Crede's silver ointment and Pryor's method—curettage and packing of the womb, and through a broad incision in the posterior cul-de-sac, of the pelvic cavity with iodoform gauze, the alleged benefits of which he attributes to breaking up of the iodoform and absorption of iodin by the blood—to abdominal hysterectomy, which has doubtless saved lives, but which I mention only to show how desperate are the straits to which we are sometimes reduced in the management of this highly-preventable disease. "Causes, causes, and again causes, more and more we fall back on these as the chief objects of our attention."—*Occidental Medical Times.*

### THE CORONER'S INQUEST A MEDIEVAL RELIC.

Among the legacies of former ages which our English ancestors brought with them to this country were the two officials, the sheriff and the coroner. Both were county officers, but the county itself as a factor in the excellent system of local self-government transmitted to our shores, is gradually becoming of less importance when compared with the stronger individual governments of the cities and towns. This is largely due to the rapidly increasing density of population and the tendency towards aggregation of the people in urban communities. At no time, especially in the New England States, has the county had the individuality which appears in the government of the town, the former having been constituted as a matter of convenience for the purpose, mainly, of facilitating judicial business and the management of roads and bridges.

The two county officers, the sheriff and the coroner, were transplanted upon our own soil in the seventeenth century. Each of these officials is as old as the days of King Edward I. of England, and possibly older; but, while the sheriff is a necessity, and has grown to be an important executive functionary—much more so in the United States than he is in England—the office of coroner, on the other hand, as conducted at the present day, requires such incongruous functions as to make it a matter of wonder that every American State has not shaken off this medieval incumbrance and adopted methods in harmony with modern progress.

During the six centuries which elapsed in England from the days of Edward I. down to the present time, far better methods were being evolved in continental countries for the purpose of conducting medico-legal investigations. But England has always been slow to adopt radical changes in forms of government, notwithstanding the fact that Scotland had discarded the coroner's iuquest many years ago.

In one of his novels ("By Order of the King,") Victor Hugo, with the keenest satire and an acute insight into the defects of some of the quaint institutions of English government, justly ridicules the coroner's inquest in the most unsparing manner. In describing some of the social clubs of the seventeenth and eighteenth centuries, he says:

"There was the Butting Club, so called from its members butting folks with their heads. They found some street porter with a wide chest and a stupid countenance. They offered him, and compelled him if necessary, to accept a pot of porter, in return for which he was to allow them to butt him with their heads four times in the chest, and on this they betted. One day a man, a great brute of a Welshman named Gogangerdd, expired at the third butt. This looked serious. An inquest was held and the jury returned the following verdict: 'Died of an inflation of the heart, caused by excessive drinking.' Gogangerdd had certainly drunk the contents of the pot of porter."

That this satirical thrust applies with equal force to the coroner's inquest of to-day may be readily shown by reference to the Parliamentary investigation of 1893, on the subject of Death Certification, page 8. The following verdict of a jury is quoted in that report: "This man died of stone in the kidney, which stone he swallowed when lying on a gravel-path in a state of drunkenness."

What then are the defects of the coroner's inquest, and how may they be remedied? The two radical defects may be summarized as follows:

1. *The combination in one person of two entirely incongruous functions*—law and medicine. The object or end of the coroner's inquest is two-fold. First, to determine the cause of death, a medical question; and second, to fix the responsibility for the death, a problem which, especially in cases of homicide, involves a knowledge of law. It is, therefore, plainly an absurdity to unite both of these functions in one person. It is true that an occasional professor of medical jurisprudence might be found who combines both branches of training; but, to expect that such persons can be found in every district requiring the services of an official empowered to investigate deaths by violence is clearly impossible.

It is not remarkable that, in the remote periods of history, when lands were sparsely settled and both medical and legal training were in their infancy, such a union of functions should be vested in one individual; but it is not creditable to the good sense of the marvelously progressive profession of medicine that it should still continue to tolerate a condition of affairs so inconsistent with modern progress. That one man should at once be familiar with all the legal complications and technicalities involved in fixing the responsibility of deaths by violence, and at the same time should be an expert in anatomy, surgery, pathology and toxicology, is absurd. He must be familiar with the effect of gun-shot and other wounds, and the pathologic changes which follow their occurrence when death follows them at a period remote from the reception of the wound. He must not only know the character of poisons, but must also be familiar with their effects upon the system and the changes which they produce in the tissues. In a word, he must be a medical expert upon the subject of the causes of death, and especially of deaths by violence. It is the glaring incongruity of attempting to successfully combine two professions in one official, that so often makes the inquest a source of ridicule.

2. *The Retention of the Jury.*—We here find a needless body of men, called upon in many instances to decide upon technical questions which can far better be settled by a single expert well trained for the purpose. The jury is not only needless, but expensive, since it involves the payment of a half dozen men (in some States twelve) for services which can be better performed by a single expert. Thousands of deaths by violence have been investigated in Germany, France and other countries in a far more thorough and satisfactory manner than is done in England and the United States, and this without the needless intervention of a jury. It is needless for the reason that, in every case of death by criminal means, a jury must finally decide the responsibility of the criminal in a court of law. Why then should two juries act in the case when one only is necessary? Another evidence of the inconsistency of the coroner's inquest is the fact that the findings of such deliberative bodies are very often the subject of ridicule in the columns of the daily press.

An illustration of the defects of the old system is shown in the noted Barron case, which occurred several years since in Maine. A bank cashier was found dead in the safe-vault of the bank. The "good-natured coroner," as was stated at the time, deferred to the wishes of the family, and no autopsy was conducted. Consequently the body was buried, and to this day the whole community is in doubt whether the cashier was murdered or committed suicide. It is safe to say that a thoroughly trained medical official, acting under an efficient law, would have insisted upon the necessity of a thorough examination, which would, in all probability, have settled the doubtful question.

How, then, shall these defects be remedied, and what difficulties stand in the way?

Abolish the coroner and his jury by an act of legislature, and substitute in their place a corps of well-trained medical experts, whose duty it shall be to investigate all deaths by violence, and then report them to designated judicial authorities for further investigation, thus separating the medical from the legal function and delegating each to its proper set of officials.

If, as is the case in the State of New York, it happens that the coroner is a constitutional officer, then it becomes necessary to amend the constitution in order to get rid of him. It may happen also in many States that the coroner has unusual political influence. In such cases the more thoroughly the incongruity of his office and the needless expense of the jury are exposed, the better for the State and the whole community. Enlightened public opinion will lend its aid in accomplishing the desired end.

As an example of the successful working of such an improved system, I need only refer to the experience of Massachusetts, which has had neither coroner nor jury for more than twenty years, during which time about 35,000 cases of violent, suspicious and sudden deaths have been investigated in the most careful and thorough manner and with less expense than was possible under the old system. The radical overturn wrought by the enactment of the law of 1877, abolishing the coroner and his jury has been one of the most satisfactory changes ever effected in the history of State legislation. Similar though not so radical changes were afterward enacted in Rhode Island and Connecticut with satisfactory results.

Further information upon this subject may be had by consulting Wood's Reference Handbook, vol. 2, articles, Coroner and Examiner; also the 45th Mass. Registration Report, 1886, Transactions of the Medico-legal Soc., vol. 1, pp. 204–208, and 28th Report of the Mass. State Board of Health, 1896, p. 817.—*Samuel W. Abbott, M.D., Boston, Mass., in Philadelphia Medical Journal.*

### MR. SPITTER OF SPITTERVILLE.

One of the small towns in a State near Minnesota will serve to illustrate the manners and customs of other towns, great and small, where the expert expectorator resides as a prince in his kingdom.

This imperious ejector is proud of his accomplishments, and keeps himself constantly before the public eye. He frequents all the resorts and public places within his domain to exhibit his prowess, his art and skill, as well as to demonstrate the strength and force of his facial ejaculatory muscles.

His presence in the office of the country hotel seems almost a village necessity. There he is in his element, listening to wondrous and boastful tales of smut and adventure, punctuating the pauses with frequent explosions of saliva, nasopharyngeal or pulmonary exudate. In his deliberation he mechanically speculates whether it would be best to spit over or under the stove, and finally compromises with his conscience by spitting directly upon it.

If by any chance he is crowded from his favorite place, he spits on the floor or the baseboard, or attempts to fill up a corner in the room with his ever-ready fluid. If frustrated in his designs he spits anywhere and everywhere, particularly showing his indignation at the bruised and ancient cuspidor by spitting a circle around it.

As the crowd at the hotel thins out, our prince repairs to the court-house where an important trial is in progress. On his journey he follows a custom established early in the morning, spitting vigorously until he reaches the court-room, where he selects a seat forward so that he can hear the witnesses, and be seen by them as he cheerfully resumes his occupation.

He sees in front of him a printed card-board sign with the words, "Please don't spit on the floor," and his indignation is unbounded. He feels that his personal liberty is about to be enjoined, and for a moment he hesitates, but on catching sight of a large spittoon, about one hundred feet distant, and to carry out the spirit if not the letter of the printed request, he skillfully aims at the receptacle, but falls short about ninety-six feet. For a moment he is astonished, but soon recoveres his equilibrium, and in his further endeavor to obey the injunction he tries again, and fails as before. By this time he begins to take an interest in the trial, and forgets the injunction as his circle of salivary explosives widens. As his interest increases the circle grows smaller, and he uses all the floor space unoccupied by his feet.

When the floor is of sufficient volume, he changes his seat, and begins anew his flooding process.

The platform occupied by the court, jury and attorneys is elevated and carpeted, and is regarded by our hero as a sacred seat of justice, filled with the dignitaries of the bar. His respect for them is unbounded, until he finds them using the carpet as a soft mark for their expectorations, and then he knows that many of them belong to the lodge of S. P. I. T. He, our prince, remembers the carpet episode, and when opportunity offers, he spits upon it with great contempt, as if to show his abilities in their fullest light.

When court adjourns he sallies out with a few genial companions, who are his ejaculatory admirers, decorating the matting on the main court-room. As the trio shamble down the stairs, each step is decorated in turn faitfully and abundantly. Should a step escape, the next member of the lodge is expected to supply the missing decoration. When out in the air again, they abandon themselves to a joyous competition, and spit on the sidewalk, spit over the sidewalk, spit between the cracks, on the fence or passing objects, and, as a further evidence of lightheartedness, they spit in the air, they spit at the wind; each spits on his shirt, each spits on his vest, and if he misses himself he spits on the rest.

When the expert finally and lazily flings himself into a vacant chair on the hotel sidewalk, he decorates and befouls the sidewalk, endangering the garments of passers-by, and laughs with glee to see a lady walk around his artificial lake of pulmonary or naso-pharyngeal excrement.

As the prince of Spitterville journeys to a neighboring settlement, the train floor receives his undivided and persistent attention.

Passengers who are otherwise inclined hail with joy (?) this welcome and highly cultivated individual, knowing that he will keep a few seats for his own purpose and defilement.

For such an individual speedy and just punishment is urged. He should be decorated with a medal in the form of a painted tin cup, tied and sealed about his neck until such time as may be decided upon by the local health authorities or until the spitter can give bonds to thereafter keep the law.

On account of this nasty, disagreeable and health-disturbing, but generally adopted habit, it is perhaps best to class it as a neuromimesis, to be treated by proper discipline and legal restraint.

It is certainly gratifying to note the marked abatement in the spitting habit in larger cities, where the caution and commands of the local boards of health, enforced by an occasional arrest, trial and fine, have taught the spitter the lesson, that it is better to observe the law of the land, as well as to follow the instructions of the health office and prevent the indiscriminate spread of disease by promiscuous expectoration.

If any one thing more than another impresses a visitor in a country town, it is the cleanliness of the people and their habitations. There is neither justice nor reason for the continuance of this spitting hibit in the country. If the local boards of health in villages and towns would join in a protest and by printed commands would warn and educate the people, the winds and dust of a prairie town would not carry the dried sputum of expectoration over the country. The health boards of the larger cities would heartily co-operate with other boards in this matter and would sign protests for distribution.—*Northwestern Lancet.*

---

### SELF-PERFORMED CÆSAREAN SECTION.

Probably nothing better shows the remarkable immunity enjoyed by the peritoneum than the interesting cases of self-performed Cæsarean section, that from time to time are reported in the current literature. Besides manifesting an astounding degree of stoicism, these patients seem to have the happy faculty of evading the disastrous consequences of their temerity, and that notwithstanding the most inauspicious circumstances under which the act is consummated. Filth to them is apparently innocuous, and bacilli have no terror.

A remarkable fact associated with these blood-curdling reports is that most, if not all, of the cases have occurred among the degraded classes of Southern and Eastern Europe, as in the last instance recorded by Loffler ( in the *Wiener Med. Woch.*, No. 10, 1901 ), the victim being a Turkish peasant woman. Suffering from some obscure chronic affection, and fearing she would perish before the termination of her pregnancy, this stoical creature, at the eighth month of gestation, deliberately opened her abdomen and uterus with an ordinary pen-knife. As the child emerged, the woman fainted from shock and loss of blood. On regaining consciousness sometime afterwards the wound was sewed up, at her request, by her daughter, a child of only thirteen years of age, an ordinary needle and waxed hemp thread being employed for the purpose. Notwithstanding these primitive measures, and the fact that a simple Cæsarean section was performed, that is, without the insertion of uterine ligatures, the woman made an uninterrupted recovery. There was no manifestations of sepsis or peritonitis, and union of the abdominal incision was unattended with suppuration. The abdominal dressing employed was a layer of moss held in place by a filthy linen cloth. The child, which also survived, was nursed by its convalescent mother.

Such cases seem to indicate the uselessness of the modern methods of antisepsis. If patients placed in the most unfavorable of circumstances can recover from the gravest of injuries without the development of any untoward symptoms, it would seem that the extreme care practiced by the modern surgeon is altogether unnecessary and a waste of valuable time and material. Such cases naturally fall in line with those remarkable instances recorded of unbroken recovery following most extensive traumatism—accidental, military and surgical.

Many feet of bowel may be resected from one individual without ill-result, while a simple enteroraphy in another will be rapidly followed by a fatal termination ; gravel, filth, and curious foreign bodies gain entrance into the peritoneal cavity and apparently excite not the slightest irritation, while a simple exploratory incision will be followed by grave or even fatal sepsis. This explanation of this curious phenomenon must be found in some refinement and extreme developmental sensitiveness of the tissues, whereby in one case there will be an apathy of the parts to external influence and in another a high degree or reaction. It is well known that individuals of higher mental and social development will react more promptly to these deleterious influences than will individuals much lower in the mental and social scale. The leader in the community will succumb to a moderately severe peritoneal operation, while the hod carrier will recover from some grave lesion without any untoward symptom to interrupt the progress of the recovery. In the auto-Cæsarean section, above recorded, and in the others that have filled the curiosity-pages of surgery, this low position in the social scale was one of the attendant features in the cases. It was not because of the lack of surgical care that recovery followed, but in spite of the dangerous concomitants of the operation.—*The Philadelphia Medical Journal.*

---

### A NEW NOSE CUP.

Paiented July 2, 1901.

This cup has been designed by W. J. Evans, New York City, to meet the demand for an instrument for washing out the nasal cavity, which could be used without fear of injury to the breathing passages, or forcing of fluid into the Eustachian tubes. It admits of the natural method of cleansing the nose, or, as it has been termed "drinking through the nose." This method is at once easily taught and easily learned, and is efficient without being harmful. The fluid is placed in the cup, the higher curve of the rim being adjusted beneath the nostrils. The cup is tilted until the liquid enters the nostrils, then, closing the mouth, a slight drawing in of the breath causes the solution to enter the nose and naso-pharynx, thus bringing it in contact with all parts of the nasal mucous membrane. The solution may be allowed to pass to the back of the throat and be expelled through the mouth. If it is desired to douche one nostril at a time, this may be easily accomplished by alternately pressing the elevated rim of the cup against the wing of the nostril, thus completely closing it. The cup can be obtained from McKesson & Robbins, New York.

## Figures and Tables

**Figure f1:**